# Supplementary material for: Stable isotopes as a predictor for organic or conventional classification of berries and vegetables
Source: PLoS One. 2025 Mar 12;20(3):e0318179. doi: 10.1371/journal.pone.0318179 (PMC11902282; doi:10.1371/journal.pone.0318179)
Supplement: S1 File — (PDF) [file pone.0318179.s001.pdf]

Supplementary for "Stable Isotopes as a Predictor for Organic or Conventional Classification of Berries and Vegetables"

Table S1. Carbon (C, %) and nitrogen (N, %) content, C/N ratio, and isotopic composition of C ($\delta^{13}C$, ‰) and N ($\delta^{15}N$, ‰) from berries and vegetables commercialized as organically and conventionally produced. Each number represents the mean value and standard deviations are given in parentheses. For each measured variable, different uppercase letters indicate a significant difference between production systems within each crop, whereas different lowercase letters indicate significant differences among geographic origin ($p < 0.05$). Letters only listed for pairs of means that are significantly different.

Country	Crop	Num.	Conventional System					Num.	Organic System				
			C	N	C/N ratio	$\delta^{13}C$	$\delta^{15}N$		C	N	C/N ratio	$\delta^{13}C$	$\delta^{15}N$
Mexico	Blackberry	41	42.52 (1.90)	1.11 (0.21)	39.68 (7.53)	-25.69 (0.89)	4.05 (1.44)B	28	42.97 (1.67)	1.10 (0.31)	41.47 (8.94)	-25.68a (0.94)	5.97 (3.16)A
	Blueberry	36	41.43 (1.04)	0.60 (0.13)a	72.83 (15.98)b	-23.90 (1.39)a	1.16 (2.06)B	19	41.13 (1.08)	0.55 (0.15)	78.81 (14.92)ab	-24.00 (1.64)a	5.35 (3.77)Aa
	Broccoli	29	43.00 (1.13)	4.13 (0.40)Bb	10.49 (0.82)Aa	-26.21 (0.62)a	3.84 (2.15)a	27	43.49 (0.74)	4.47 (0.43)A	9.80 (0.87)B	-26.26 (0.59)a	4.07 (2.40)b
	Celery	2	35.73 (0.41)	1.45 0.50-)b	26.16 (8.73) a	-27.85 (0.34)	2.89 (1.88)	38	35.08 (1.03)	1.51 (0.29)	24.11 (5.22)b	-27.24 (0.63)	5.97 (2.21) b
	Lettuce	3	40.53 (0.68)	3.65 (0.15)	11.12 (0.61)	-25.51 (0.52)	3.19 (6.46)B	32	40.22 (0.57)	3.23 (0.47)	12.76 (2.14)	-25.85 (0.62)	8.40 (1.87)A
	Raspberry	26	43.54 (1.88)	1.21 (0.19)	36.75 (6.01)	-25.87 (1.21)	1.71 (1.56)B	33	44.12 (1.57)	1.33 (0.68)	37.31 (10.18)	-26.28 (1.02)	6.91 (2.50) A
	Strawberry	30	40.84 (0.99)a	1.26 (0.21)a	33.00 (4.73)b	-24.96 (1.08)A	2.24 (1.79)Ba	36	40.76 (0.60)	1.18 (0.23)	36.18 (9.22)	-25.60 (0.60)Bb	7.39 (2.88)Aa
U.S.	Blackberry	4	40.81 (0.97)B	1.12 (0.12)	36.68 (3.74)	-26.20 (0.43)	2.66 (1.61)B	7	44.09 (1.28)A	1.07 (0.27)	43.12 (9.07)	-26.71 (1.22)b	5.25 (1.87)A
	Blueberry	31	41.70 (0.83)A	0.57 (0.15)a	77.33 (19.46)b	-26.59 (1.24)b	0.49 (2.40)B	35	41.11 (0.73)B	0.59 (0.12)	72.73 (14.48)b	-25.97 (1.49)b	5.09 (2.84)Aa
	Broccoli	33	42.99 (1.08)	5.16 (0.44)Aa	8.40 (0.84)b	-27.86 (1.12)b	1.91 (1.79)Bb	25	43.08 (0.81)	4.71 (1.01)B	13.85 (24.82)	-27.88 (0.99)b	7.10 (2.67)Aa
	Celery	30	35.47 (1.41)	2.22 (0.41)Aa	16.57 (3.75)Bb	-28.18 (0.78)B	1.67 (2.79)B	31	35.54 (1.06)	1.36 (0.39)B	28.19 (8.16)Aa	-27.56 (0.89)A	9.85 (2.74)Aa
	Lettuce	32	40.29 (1.53)	3.47 (0.42)	11.76 (1.27)	-26.39 (0.72)	0.28 (1.94)B	31	40.61 (0.94)	3.51 (0.42)	11.75 (1.55)	-26.49 (0.91)	7.03 (2.95)A
	Raspberry	6	43.98 (1.76)	1.12 (0.21)	40.28 (6.69)	-26.16 (1.59)	0.92 (1.41)B	1	43.20 (-)	1.24 (-)	34.76 (-)	-26.99 (-)	12.07 (-) A
	Strawberry	30	40.35 (0.80)Bb	1.15 (0.14)b	35.95 (6.41)a	-24.86 (0.90)	1.42 (1.32)Bb	32	40.89 (0.84)A	1.08 (0.18)	39.00 (6.80)	-24.88 (0.74)a	5.12 (1.65)Ab
Others	Blackberry							1	41.35 (-)	0.95 (-)	43.66 (-)	-26.82 (-)ab	4.36 (-)
	Blueberry	33	41.67 (1.25)	0.47 (0.19)b	99.52 (34.90)a	-26.54 (1.43)b	1.33 (1.02)A	39	41.80 (1.24)	0.52 (0.17)	88.10 (25.80)a	-27.16 (1.25)c	0.65 (1.69)Bb
	Broccoli	3	43.84 (0.44)	4.80 (0.16)a	9.14 (0.22)b	-28.04 (1.39)b	0.27 (1.08)b	4	43.92 (0.59)	4.90 (0.22)	8.98 (0.31)	-29.29 (1.18)c	2.37 (1.53)b
	Celery							1	35.23 (-)	1.79 (-)	19.70 (-)ab	-28.68 (-)	4.97 (-)ab
	Raspberry	1	40.09 (-)	1.45 (-)	27.63 (-)	-24.54 (-)	2.77 (-)						
	Strawberry							1	40.94 (-)	1.20 (-)	34.08 (-)	-25.76 (-)ab	5.08 (-)ab

Table S2: Table shows relative importance of variables in the final model in order of (Rao) score statistic. All variables are considered fixed in this model, and each row represents a reduced model with the variable removed from the full model.

Variable*	Residual Dev.	Model df	Test df	Dev. Difference	Score Statistic	$p^{\dagger}$
Full Model	249.4	663				
Company	657.8	769	106	408.3	294.6	1.12e-19
$\delta^{15}N$	518.4	670	7	268.9	168.5	5.33e-33
CN Ratio	291.4	670	7	41.9	35.9	7.65e-06
Product	350.5	681	18	101.1	61.3	1.27e-06
C %	258.7	664	1	9.2	8.9	2.84e-03

*variables are dropped from the full model along with higher order terms
†*p*-values are based on the score statistic

Table S3: PA coefficients of the final GLMM model, split into each crop. The binary response (organic = 1, conventional = 0) is linked to the covariates by the logit function, implying that the slope coefficients are on the log-odds scale. *p*-values are testing whether the coefficient is 0, with a two-sided alternative by normal approximation with robust standard errors.

	intercept	C %	C/N ratio	$\delta^{15}N$
Blueberry	-5.66*	0.09+	0.01*	0.12**
Broccoli	-5.66*	0.09+	0.28	0.16**
Celery	-5.66*	0.09+	0.04	0.30***
Raspberry	-5.66*	0.09+	-0.30***	3.36***
Romaine Lettuce	-5.66*	0.09+	0.04	0.35
Strawberry	-5.66*	0.09+	0.08+	0.80***
Blackberry	-5.66*	0.09+	0.01	0.18**

Significance coding: '<0.001' = ***, '<0.01' = **, '<0.05' = *, '<0.1' = +, and no symbol otherwise.

Table S4: Classification performance of the final PA GLMM for each crop. The classifications are made by thresholding the predicted values from the regression model. Confidence intervals were bootstrapped with 2000 replicates around each threshold point to give 95% confidence of <1% FNR. The model shows the best overall performance for Romaine lettuce, raspberries, celery, and strawberries. Blueberry and blackberries show less accuracy and specificity, implying stronger tradeoffs for controlling the FNR.

Product	Threshold*	Specificity†	Accuracy†	AUROC‡
Blueberry	0.22	0.00	0.48	0.69
Broccoli	0.27	0.14	0.54	0.72
Blackberry	0.19	0.04	0.47	0.73
Romaine Lettuce	0.24	0.63	0.87	0.97
Strawberry	0.05	0.17	0.61	0.94
Raspberry	0.31	0.85	0.93	0.98
Celery	0.19	0.53	0.85	0.94

*Thresholds are chosen to give 95% confidence of < 1% FNR for each product.

†Specificity and Accuracy are at the given Threshold value.

‡Area under the Receiver Operating Characteristic for population averaged predictions.

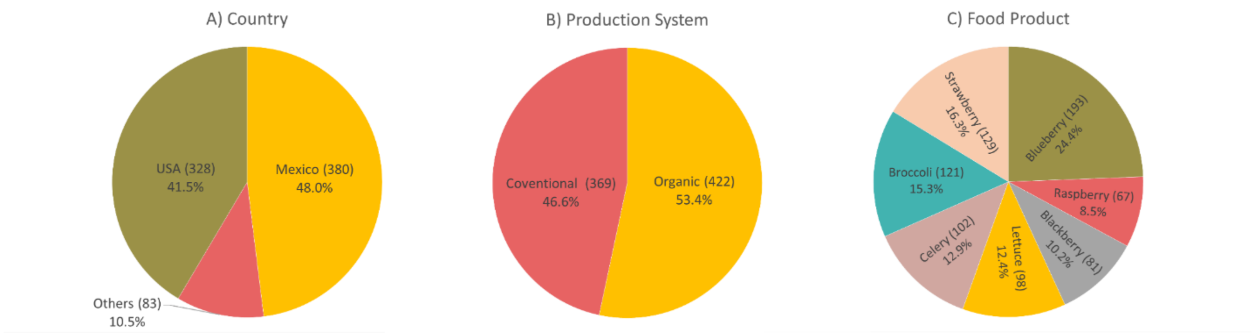

Fig. S1 Distribution of the fresh produce samples collected by country of origin (A), production system (B), and type of crop (C).

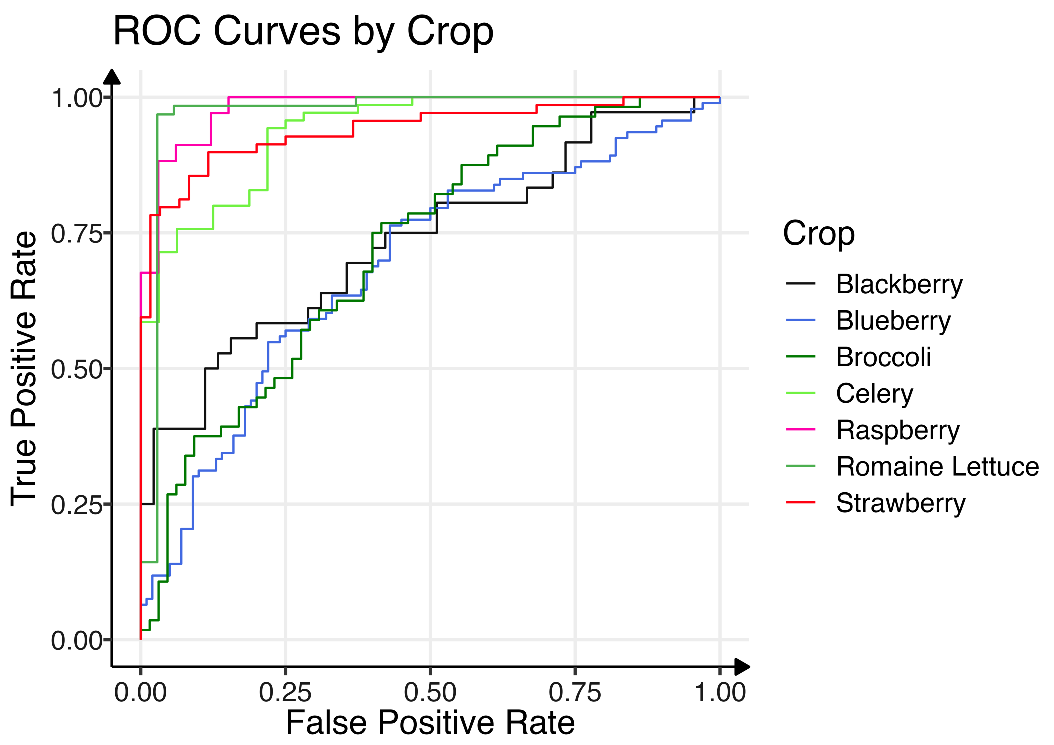

Fig. S2: Raspberries, Romaine lettuce, strawberries and celery perform the best overall (by area under the ROC curve) in the final PA GLMM model. In order to maintain a low false negative rate (high TPR), we restrict ourselves to the upper section of the plot and note that raspberries have the lowest FPR while also maintaining a high TPR.
