# Supplementary material for: Stable isotopes as a predictor for organic or conventional classification of berries and vegetables
Source: PLoS One. 2025 Mar 12;20(3):e0318179. doi: 10.1371/journal.pone.0318179 (PMC11902282; doi:10.1371/journal.pone.0318179)
Supplement: Attachment — Submitted filename: Responses to Reviewers.docx [file pone.0318179.s004.docx]

*Dear Editor,*

*We have uploaded a revised manuscript that takes into consideration the reviewer comments which we found to be constructive and helpful. We have also uploaded a response letter that detailed our point-by-point responses to the reviewers’ comments with references to line numbers in the revised version. Let me know if further information or modifications are needed.*

*Best wishes,*

Reviewers’ Comments to the Author

Reviewer #1: The manuscript is well-designed and well-prepared. only some comments in the attached reviewed manuscript. Also the manuscript could be very benefits from an English editing to improve the flow of the text and rephrasing some sentences

*We thank the reviewer for the positive feedback. We have now addressed the comments that reviewer has indicated in the manuscript.*

1. Line 51-54: Rephrase this sentence

*We have rephrased this sentence to “Among these challenges, organic farming systems navigate the complexities introduced by the prohibition of synthetic inputs, which adds layers of management difficulty and economical constraints that are not typically encountered in conventional production systems ”. See Line 61-63 of the revised manuscript.*

2. Line 63: Add the reference: Alneyadi, K., Almheiri, M. S. B., Tzortzakis, N., Di Gioia, F., & Ahmed, Z. F. R. (2024). Organic-based nutrient solutions for sustainable vegetable production in a zero-runoff soilless growing system. Journal of Agriculture and Food Research, 101035.

*We have cited the reference in the line 90 of the revised manuscript*

Reviewer #2: Dear Authors,
Thank you for the opportunity to contribute to your work as a reviewer. This study aims to introduce a methodology for the differentiation of organic and non-organic produces with the quantification of N stable isotopes. The experiment is well designed and the results are visualized in an understandable way. However, clarifications needed when interpreting the results, and there is space for further explanations.

*We sincerely appreciate the time and effort the reviewer has invested in reviewing our manuscript. Your willingness to navigate the complexities of this work and provide detailed, constructive feedback is incredibly valuable to us. We are grateful for the opportunity to learn from your insights and suggestions for improvement. Thank you for contributing to the refinement of our research with your expertise and thoughtful analysis.*

General comments
1. It is a common misbelief, that organic farming is organic just because of the fact, that the farmers do not use chemicals. Instead, it is organic, because the certification body says, based on the regulatory background, that it is organic. Therefore, it is not clear, what the sentence in line 19 is about – please revise. Also, it would be useful to add a clarifying sentence about the USDA/EU regulatory background on organics.

*Thank you for your observation. We agree that organic certification is determined by compliance with specific regulatory standards set by USDA/EU frameworks, rather than merely the absence of synthetic chemicals. The revised sentence clarifies that while certification bodies do validate adherence to organic standards, there remains limited on-ground verification to ensure that all practices—such as the use of organic fertilizer sources—are continuously followed. Additionally, we have included a sentence summarizing the USDA/EU regulatory framework as suggested. See lines 21- 25 of the revised manuscript.*

2. Regarding the collection of samples, it is unclear for me, when did the authors purchased the vegetables and berries. It is reported, that it took a whole year, but no information is available about the exact time or the production system those were grown. All of the samples were grown on genuine soil? Were those from uncontrolled/controlled environment facilities, hydroponics, aeroponics, etc? Were those fresh or stored products? And, most importantly, within species, were those purchased in more or less the same time? Obviously, when comparing a CEA lettuce harvested in January and an open-field one harvested in October does not seem fair. At the same time, I would like to emphasize, that I do not doubt the results due to the high number of samples, but I find it important to add more general information about the origin of the produces (not detailed information one by one!) and clarifying the possible issues arising from that.

*Samples were collected continuously from late January to early September across the specified counties in California, based on availability. This approach ensured the sample pool represented a broad range of suppliers, including both growers and retailers. Note that sampling did not occur during the cool season when greenhouse production is more prevalent, though still uncommon for the crops of interest. It is important to acknowledge that we could not determine whether individual produce items were grown in greenhouses, open fields, or soilless cultures. Based on our knowledge and discussions with growers on the US West Coast and in Mexico, berries are frequently cultivated in open greenhouses (ambiently vented). However, lettuce, celery, and strawberries found in the California market from February to September are highly unlikely to come from soilless culture facilities due to the economic disadvantage of competing with field-grown produce from the western US and northern Mexico, where most of our samples originated. Overall, we believe that differences in growing conditions did not significantly impact our authenticity evaluation, particularly given that the vast majority (>95%) of samples were field-grown in the US and Mexico. The uniformity of delta-13C values within each type of produce further suggests field cultivation, as greenhouse-grown samples can exhibit altered 13C values due to CO2 from fuel combustion or CO2 enrichment, which can drastically lower delta-13C values. Additionally, only fresh samples were purchased—no frozen or processed items were included. Samples were frozen immediately after purchase and stored in a freezer until processing in the lab.*

3. The Authors mention, that this method can be used to demonstrate unauthorized use of synthetic fertilizers in OF. What is the amount they think it is possible to detect the difference between OF and non-OF products? I am asking it as the results showed, that the most robust difference was found in the case of lettuce, which is known to be produced with the use of high doses of synthetic fertilizer in conventional farming, therefore the difference can be high comparing with OF.

*We appreciate the reviewer’s excellent question. At this stage, our study does not allow us to determine a precise threshold for detecting unauthorized synthetic fertilizer use in organic farming (OF). However, this research serves as a foundational step, providing data that future studies can build upon. To establish detection limits, controlled experiments will be necessary to investigate how varying levels of synthetic and organic fertilizer integration affect the δ¹⁵N signature of crop products. This will allow us to further refine methods for distinguishing between OF and non-OF products based on δ¹⁵N.*

4. I am unsure whether the differentiation is about OF versus non-OF, as the farmer should not be a certified organic farmer for the use of manure or compost. Again, organic is about the certification provided by the authorization body. The authors might want to deal briefly with this question in the ms. Additionally, as the breakdown products of any chemicals used in the field remain in the soil and might travel with soil water, OF produces can also be polluted with these materials, without breaking the regulation, especially in areas where heavy agrochemization is an issue.

*We appreciate the reviewer’s insightful comment. It is true that the use of manure or compost alone does not qualify a farm as certified organic; organic certification requires compliance with standards set by the authorizing body. However, the use of manure or compost typically results in higher δ¹⁵N values in produce than if no organic fertilizers were used. In conventional farming, while manure or compost may be used, synthetic fertilizers are generally applied extensively, resulting in a δ¹⁵N signature that is often lower than in produce relying solely on organic fertilizers. Therefore, in our manuscript, we acknowledge that some conventional produce may exhibit higher δ¹⁵N values. However, we would not expect δ¹⁵N signatures of certified organic produce to fall below those of their conventional counterparts, given that organic farming prohibits synthetic fertilizers. We also recognize that contamination from adjacent conventional fields could contribute to δ¹⁵N values, potentially diminishing the differences between organic and conventional fertilizers, but would not decreasing δ¹⁵N signatures of certified organic produce to fall below those of their conventional counterparts. So, δ¹⁵N can still be used to differentiate organic produce from their conventional counterparts, especially flagging out organic produce with extremely low δ¹⁵N values that require further investigation (Line 317-320 of the revised manuscript) . Our study represents a first step in developing a market-based dataset to examine these systemic differences and test the concept of distinguishing organic and conventional produce by δ¹⁵N signature. The results provide a foundation for future studies to explore factors influencing δ¹⁵N in produce and to investigate potential causes for insignificant differences in certain cases. See lines 364-411 of the revised manuscript for detailed discussion.*

Detailed comments
line 13: significantly?

*Revised.*

line 47: What about the EU? Or add: For example, (…)

*We revised the sentence to “For produce to be certified organic, such as in the U.S., it must be grown without synthetic fertilizer and pesticide inputs…”. See lines 55-58 of the revised manuscript.*

line 49: It is not the certification process, that leads to higher production costs, but the exclusion of cheap agrochemicals. Please revise.

*We revised the sentence to “The exclusion of conventional agrochemicals in organic farming often leads to higher production costs compared to conventional farming, primarily due to lower yields… ”. See lines 58-60 of the revised manuscript.*

line 54: Revise the sentence starting with „This prohibition …

*We revised the sentence to “This prohibition poses a particular challenge in aligning nitrogen (N) availability from organic sources with the timing of crop demand. See lines 63-65 of the revised manuscript.*

line 84-85: Revise.

*We revised to “This makes it possible to use the δ^15^N value of produce to indicate the specific N-based fertilizing material used in a cropping system.” See lines 109-110 of the revised manuscript.*

line 120: Please use complete scientific names, or at least add sp.

*We agree. We revised the names to scientific names for the produce: strawberry (Fragaria × ananassa), blueberry (Vaccinium sect. Cyanococus), blackberry (Rubus subg. Rubus), and raspberry (Rubus idaeus)) and vegetables (broccoli (Brassica oleracea var. italica), celery (Apium graveolens), and lettuce (Lactuca sativa var. longifolia)” (line 167-170 of the revised manuscript).*

line 263: Was that romaine lettuce, or any type of lettuce? Clarify throughout the whole ms.

*Yes, we sampled only romaine lettuce for this study. Since the scientific name Lactuca sativa var. longifolia specifically refers to romaine lettuce, we have used 'lettuce' instead of 'romaine lettuce' throughout the manuscript to avoid redundance (line 331 of the revised manuscript).*

line 269: ’Company’ within inverted commas?

*We have added inverted commas over company in the revised manuscript (Line 339 of the revised manuscript)*

line 295: There are several factors besides climate and soil conditions, that counts, please add more.

*We agree that many additional factors contribute to variations in the δ¹⁵N signature in produce, including land management history, geological region, atmospheric deposition, microbial activity, types of organic fertilizer, irrigation practices, tillage practices, and biological nitrogen fixation. Among these factors, geological region and atmospheric deposition are often associated with climate regions, while microbial activity, biological nitrogen fixation, and land management history are closely related to soil conditions. Therefore, in the revised manuscript, we have incorporated additional considerations for land management practices and sources of organic fertilizer, as well as discussed how these factors impact δ¹⁵N signature in produce. See lines 364-411 of the revised manuscript.*

line 307: Slovenian?

*Revised. See line 420 of the revised manuscript.*
